# Moving Forward by Stimulating the Brain: Transcranial Direct Current Stimulation in Post-Stroke Hemiparesis

**DOI:** 10.3389/fnhum.2016.00394

**Published:** 2016-08-09

**Authors:** Heather T. Peters, Dylan J. Edwards, Susan Wortman-Jutt, Stephen J. Page

**Affiliations:** ^1^Division of Occupational Therapy, The Ohio State UniversityColumbus, OH, USA; ^2^Better Rehabilitation and Assessment for Improved Neuro-recovery (B.R.A.I.N.) Laboratory, Ohio State UniversityColumbus, OH, USA; ^3^Laboratory for Non-Invasive Brain Stimulation and Human Motor Control, The Burke Medical Research InstituteWhite Plains, NY, USA; ^4^Department of Neurology, Weill Cornell Medical CollegeWhite Plains, NY, USA; ^5^Burke Rehabilitation HospitalWhite Plains, NY, USA

**Keywords:** stroke, rehabilitation, hemiparesis, transcranial direct current stimulation, non-invasive brain stimulation, neuroplasticity

## Abstract

Stroke remains a leading cause of disability worldwide, with a majority of survivors experiencing long term decrements in motor function that severely undermine quality of life. While many treatment approaches and adjunctive strategies exist to remediate motor impairment, many are only efficacious or feasible for survivors with active hand and wrist function, a population who constitute only a minority of stroke survivors. Transcranial direct current stimulation (tDCS), a type of non-invasive brain stimulation, has been increasingly utilized to increase motor function following stroke as it is able to be used with stroke survivors of varying impairment levels, is portable, is relatively inexpensive and has few side effects and contraindications. Accordingly, in recent years the number of studies investigating its efficacy when utilized as an adjunct to motor rehabilitation regimens has drastically increased. While many of these trials have reported positive and promising efficacy, methodologies vary greatly between studies, including differences in stimulation parameters, outcome measures and the nature of physical practice. As such, an urgent need remains, centering on the need to investigate these methodological differences and synthesize the most current evidence surrounding the application of tDCS for post-stroke motor rehabilitation. Accordingly, the purpose of this paper is to provide a detailed overview of the most recent tDCS literature (published 2014-2015), while highlighting these variations in methodological approach, as well to elucidate the mechanisms associated with tDCS and post-stroke motor re-learning and neuroplasticity.

Many stroke survivors exhibit hemiparesis, which undermines independence and quality of life (Mayo et al., 2002). Several rehabilitative approaches targeting hemiparetic limbs have been developed (Page et al., 2013a,b) with most incorporating task-specific physical practice of the paretic limb. These approaches attempt to exploit surviving brain tissue and, specifically, to modulate synaptic networks and strengthen connections subserving these networks. While such regimens demonstrate promise, these brain processes can be difficult to modulate with a high precision and consistency when physical practice is the singular method applied.

The therapeutic application of electrical currents to the brain has been documented for centuries (Nitsche et al., 2008), with its use becoming more clinically-plausible through the introduction of the electrical battery in the eighteenth century. In subsequent decades, brain stimulation was successfully applied for a variety of psychological disorders (Kubera et al., 2015; Xie et al., 2015), as well as pain (Ma et al., 2015). More recently, transcranial direct current stimulation (tDCS)—non-invasive brain stimulation administering a constant, low current—has been used to facilitate neurophysiological (Nitsche and Paulus, 2000) and motor changes in post-stroke hemiparesis (Butler et al., 2013). tDCS offers the additional advantage of being portable, relatively inexpensive, and straightforward in administration. As a treatment for targeting post-stroke motor impairments, tDCS can enhance or suppress brain excitability with great focality and over prolonged periods, which constitutes decided benefits over physical therapy alone.

The continued prevalence and impact of stroke-induced hemiparesis suggests a need to evaluate the efficacy of tDCS as an adjuvant to physical therapy for post-stroke motor rehabilitation. In recent years, the number of studies utilizing tDCS alongside a variety of physical rehabilitation regimens has drastically increased. While results from these trials are promising, methodologies vary greatly in terms of the nature of physical practice, stimulation montage and type of outcome measures, among other important factors. There is an urgent need to highlight these methodological differences and provide a snapshot of the most current evidence surrounding the application of tDCS for post-stroke motor rehabilitation. This paper provides an overview of recent tDCS literature (published 2014-2015), with an emphasis on methodological approach, as well a description of the mechanisms associated with tDCS and post-stroke motor re-learning and neuroplasticity.

## tDCS mechanisms

tDCS involves the application of a low-intensity direct current (DC) (typically 1–2 mA), typically delivered through two electrodes situated inside saline-soaked sponges. These currents can promote subthreshold depolarization or hyperpolarization of underlying tissue using anodal or cathodal approaches, respectively. This subthreshold modulation is achieved through electrode placement based on EEG mapped sites, with current flowing in from the anodal electrode and out via the cathodal electrode. Despite only a portion of the current actually penetrating the scalp into the cortex, tDCS induces cortical excitability changes that outlast the stimulus period (Nitsche and Paulus, 2000; Stagg and Nitsche, 2011). Indeed, neurophysiological studies using transcranial magnetic stimulation (TMS) have demonstrated corticospinal excitability changes of up to 40% using both cathodal and anodal tDCS, which can be manipulated by altering the stimulation duration and intensity (Nitsche and Paulus, 2000).

The application of tDCS to post-stroke motor recovery is based on the premise that the maladaptive interhemispheric interactions occurring post-ictus impede motor function (Murase et al., 2004). Movements of the paretic extremities are associated with high levels of transcallosal inhibition of the affected hemisphere by the less affected hemisphere (Murase et al., 2004; Takeuchi et al., 2012). Studies using multimodal imaging/mapping techniques (e.g., functional magnetic resonance imaging, TMS) have confirmed the existence of this imbalance, particularly during the preparation for and execution of skilled upper extremity (UE) movement attempts (Alagona et al., 2003; Murase et al., 2007). The existence of this contralesional inhibitory influence is associated with greater severity of impairment and poorer rehabilitative outcomes (Talelli et al., 2006). tDCS is thought to directly remediate this imbalance through upregulation of the affected hemisphere (anodal stimulation), downregulation of the un-affected hemisphere (cathodal stimulation), or use of both approaches simultaneously (bihemispheric stimulation). Although many different electrode montages are used, the two most common approaches, anodal and cathodal, will be the focus of this discussion.

## Anodal and cathodal stimulation

Stroke survivors exhibit significantly decreased ipsilesional corticospinal activity, due to damage to cortical tissue and descending corticospinal tract fibers, as well as hyper-inhibitory signals from the contralesional hemisphere (Alagona et al., 2003; Murase et al., 2004, 2007; Stinear et al., 2006; Talelli et al., 2006; Takeuchi et al., 2012). Anodal tDCS addresses these dysfunctions by causing subthreshold depolarizations of underlying membrane potential in the affected hemisphere, which may increase synaptic efficacy and response to neurorehabilitative therapies. In particular, it is hypothesized that the effects of anodal stimulation are facilitated by modulation of sodium (Na^+^) and calcium (Ca^2+^) ion channels, NMDA receptors and Gamma-Aminobutyric acid (GABA)-ergic interneurons (Nitsche et al., 2003, 2005; Stagg and Nitsche, 2011). For example, transient cortical excitability increases elicited by anodal stimulation are eliminated when subjects were given Na^+^ and Ca^2+^ ion channel blockers (Nitsche et al., 2003). Further, when given an NMDA receptor antagonist, short-term excitability was unaffected but long-term effects were eliminated. Contrarily, administration of an NMDA receptor agonist prolonged the duration of these effects. Studies using TMS (Nitsche et al., 2005) and magnetic resonance spectroscopy (Stagg et al., 2009) indicate that inhibition of GABA-ergic inter-neuronal activity is also involved and likely aids in the facilitation of these aftereffects. Taken together, modulation of ion channels and activation of NMDA receptors coupled with inhibition of GABA-ergic tone may be integral in facilitating the long-term effects of tDCS (Nitsche et al., 2003, 2005; Stagg et al., 2009; Stagg and Nitsche, 2011).

Activity in the contralesional M1 can also be downregulated via cathodal stimulation, reducing interhemispheric inhibition that is commonly observed post-stroke. Similar to anodal stimulation, there is evidence that the aftereffects of cathodal tDCS are dependent on modulation of NMDA receptors as well as glutamatergic synapses and interneurons (Nitsche et al., 2003, 2005; Stagg and Nitsche, 2011). Specifically, the aftereffects of cathodal tDCS were eliminated when subjects were given an NMDA receptor antagonist (Nitsche et al., 2003). Unlike anodal stimulation though, the administration of Na^+^ and Ca^2+^ ion channel blockers had no effect on cathodal aftereffects (Nitsche et al., 2003). In summary, there is evidence that the long-term aftereffects of cathodal stimulation are reliant on modulation of NMDA receptors, glutamatergic synapses and interneurons, but additional research is needed to determine whether or not modulation of ion channels contributes to these effects.

## Combining tDCS and motor rehabilitation

Promoting post-stroke neuroplasticity is a fundamental rehabilitative goal and the foundation on which contemporary motor rehabilitative therapies are based (Nudo, 2006; Kleim and Jones, 2008). This reorganization is thought to be experience and task-dependent, with greater reorganization occurring as a result of repetitive task practice (Nudo and Friel, 1999; Nudo, 2006). Accordingly, approaches like tDCS that directly exploit surviving brain tissue while allowing for repetitive task practice have the potential to significantly augment motor learning and outcomes.

Long-term potentiation (LTP) (long-term increase in synaptic efficiency) and long-term depression (LTD) (long-term synaptic weakening)-like mechanisms are strongly implicated as the primary factors underlying learning and plasticity in the motor cortex and may explain tDCS aftereffects (Rioult-Pedotti et al., 2000). This is because LTP and LTD also occur through modulation of NMDA receptors. Anodal tDCS, in particular, may activate these NMDA receptors, potentially resulting in a significant increase in Ca^2+^ in the post-synaptic cell (Stagg et al., 2009). As such, it is hypothesized that the motor gains observed with repeated tDCS may occur as a result of an additive effect on post-synaptic Ca^+^ levels, which lead to long-term changes in synaptic efficiency (Stagg et al., 2009). These effects on Ca^+^ ion channels and NMDA receptors may be reliant on concurrent reduction in GABA-ergic tone, which is also facilitated by anodal tDCS (Nitsche et al., 2003, 2005; Stagg et al., 2009). Given that these effects may be additive when applied repetitively and/or combined with motor training, tDCS may constitute a powerful tool in motor neurorehabilitation.

As a result of the above processes, tDCS and repetitive UE motor practice have separately been shown to improve motor function in stroke (Bastani and Jaberzadeh, 2012; Butler et al., 2013). However, the effects of tDCS alone are short lived (Nitsche et al., 2007), while physical practice alone results in only modest improvements (Young and Forster, 2007). The rationale for combining motor training and tDCS is that the effects will be additive and/or that tDCS will prime neural circuits involved in training, but the mechanisms and efficacy of such regimens require further study. Evidence in humans and non-human primates suggests effects may vary according to whether skilled or simple movements are trained, and the degree of fatigue (Plautz et al., 2000; Benwell et al., 2005; Koeneke et al., 2006), which can differentially affect antagonist-agonist muscle excitability in the stimulation target (Giacobbe et al., 2011). Nevertheless, many studies attest to the effectiveness of combined tDCS and rehabilitation in stroke, despite a substantial variation in intervention methods and dosages. Systematic analyses of the literature for tDCS in motor systems of stroke survivors indicate both favorable outcomes (Bastani and Jaberzadeh, 2012; Butler et al., 2013) and low evidence of effect (Elsner et al., 2013). A crucial difference in more recent work is the high proportion of papers reporting tDCS in combination with motor training, whereas early studies typically applied tDCS alone. This review therefore meets an important need by synthesizing the most recent work to assess the applicability of tDCS to clinical rehabilitation.

In Table 1 we present recent studies published in 2014-2015 (Au-Yeung et al., 2014; Cha et al., 2014; Fusco et al., 2014; Kim et al., 2014; Lee and Chun, 2014; Lefebvre et al., 2014; Middleton et al., 2014; O'Shea et al., 2014; Tahtis et al., 2014; Viana et al., 2014; Chang et al., 2015; Dumont et al., 2015; Gillick et al., 2015; Park et al., 2015; Rocha et al., 2015; Saeys et al., 2015; Sattler et al., 2015; Triccas et al., 2015; Yao et al., 2015; Zheng and Schlaug, 2015) that combine tDCS with post-stroke motor practice. Stimulation parameters ranged from 0.7 to 2 mA for 10–40 min, and included anodal, cathodal, and bilateral electrode configurations. The number of sessions ranged from 1 to 28, with the motor training duration ranging from 13 min to 6 h, and a larger number of studies targeting the paretic UE than the lower extremity. Of the 26 studies included in this review, 15 were randomized controlled trials, 4 were non-randomized controlled trials, 3 were randomized crossover trials, 3 were case series and 1 was a case study.

**Table 1:** **Contemporary methods of tDCS for stroke motor rehabilitation**.

**Publication**	**Extremity**	**Recovery phase**	**Number of sessions (#x) and days per week (DPW)**	**Anodal (A) Cathodal (C) Bilateral (B) Sham (S)**	**tDCS duration min**	**tDCS intensity (mA)**	**Training (Tx)**	**Sequence of tDCS and Tx**	**Effect**	**Study design**
Ang et al., 2015 (*n* = 19)	Upper	Chronic	10x 5 DPW weekdays only	B, S	20 min	1.0	60 min Motor imagery-BCI and robotics	Before Tx	⇑	RCT
Bolognini et al., 2015 (*n* = 12)	Upper	Chronic	3x alternating days	A, S	10 min	2.0	Functional hand tasks	Before and after Tx	⇑	NRCT
Cho and Cha, 2015 (*n* = 27)	Upper	Chronic	18x 3 DPW	A, S	20 min	2.0	20 min Mirror Tx for hand	During Tx	⇑	RCT
Gillick et al., 2015 (*n* = 11)	Upper	Congenital (child)	1x	B, S	10 min	0.7	n/a	n/a	⇑	RCT
Kasashima-Shindo et al., 2015 (*n* = 18)	Upper	Chronic	10x 5 DPW weekdays only	A	10 min	1.0	45 min Robotics	Before Tx	⇑	NRCT
Rocha et al., 2015 (*n* = 21)	Upper	Chronic	28x 7 DPW	A, C, S	13 min A 9 min C	1.0	6 h mCIMT	DuringTx	⇑A – C	RCT
Sattler et al., 2015 (*n* = 20)	Upper	Acute	5x 5 DPW	A, S	13 min	1.2	13 min rPNS	Before Tx	⇑	RCT
Yao et al., 2015 (*n* = 9)	Upper	Chronic	3x consecutive days	A, C, S	15 min	0.8	n/a	n/a	⇑	Case series
Chang et al., 2015 (*n* = 24)	Lower	Acute	10x 5 DPW weekdays only	A, S	10 min	2.0	2.5 h. Movement Tx	During first 10 min	⇑ Motor – Gait and balance	RCT
Dumont et al., 2015 (*n* = 1)	Lower	Chronic	1x	A	20 min	2.0	20 min Treadmill gait training	During Tx	⇑	Case study
Park et al., 2015 (*n* = 24)	Lower	Chronic	12x 3 DPW	A, S	15 min	2.0	30 min Gait training	During first 15 min Tx	⇑	RCT
Triccas et al., 2015 (*n* = 22)	Upper	Sub-acute and chronic	18x 2-3 DPW	A, S	20 min	1.0	1 h robotic training	During first 20 min Tx	–	RCT
Zheng and Schlaug, 2015 (*n* = 16)	Upper, Lower	Chronic	10x 5 DPW Weekdays Only	B	30 min	1.5	60 min customized PT/OT	During Tx	⇑	NRCT
Au-Yeung et al., 2014 (*n* = 10)	Upper	Chronic	3x Consecutive Days	A, C, S	20 min	1.0	n/a	n/a	⇑ C – A	RCOT
Lefebvre et al. 2014	Upper	Chronic	1x	B, S	20 min	1.0	n/a	n/a	⇑	RCOT
Fusco et al., 2014 (*n* = 16)	Upper	Acute	2x Consecutive Days 1x A 1x S	A, S	15 min	1.5	60 min Customized PT	Before Tx	⇑	RCT
Kim et al., 2014 (*n* = 30) 15 Healthy 15 Stroke	Upper	Subacute	1x	A	20 min	1.0	15 min Wrist Tx or Wrist Tx + VR	Before Tx	⇑	NRCT
Lee and Chun, 2014 (*n* = 59)	Upper	Subacute	15x Weekdays Only	C, S	20 min	2.0	30 min Hand/Arm OT or Hand/Arm VR	Before and during	⇑	RCT
Middleton et al., 2014 (*n* = 5)	Upper	Chronic	24x 3 DPW	B	15 min	1.5	40 min Strength and Functional Tx	During	⇑	Case series
O'Shea et al., 2014 (*n* = 13)	Upper	Chronic	1x	A, C, B, S	20 min	1.0	n/a	n/a	⇑C ⇑A – B	Case series
Viana et al., 2014 (*n* = 20)	Upper	Chronic	15x 3DPW	A, S	13 min	2.0	60 min VR	Before Tx	⇑	RCT
Saeys et al., 2015 (*n* = 31)	Lower	Subacute	16x 4 DPW Weekdays Only	B, S	20 min	1.5	60 min customized PT/OT	Before Tx	⇑	RCOT
Tahtis et al., 2014 (*n* = 14)	Lower	Subacute	14x Unknown DPW	B, S	15 min	2.0	n/a	n/a	⇑	RCT
Cha et al., 2014 (*n* = 20)	Upper and lower	Chronic	20x 5 DPW	A	20 min	1.0	30 min Functional upper and lower extremity Tx	Before Tx	⇑	RCT
Fusco et al., 2014 (*n* = 11)	Upper and lower	Subacute	10x 5 DPW Weekdays Only	C, S	10 min	1.5	45 min customized PT/OT	Before Tx	–	RCT

*⇑, Positive Effect; –, Null Effect; VR, Virtual Reality Training; OT/PT, Occupational/Physical Therapy; BCI, Brain Computer Interface; mCIMT, Modified Constraint Induced Movement Therapy; rPNS, Repetive Peripheral Nerve Stimulation; RCT, Randomized Controlled Trial; NRCT, Non-Randomized Controlled Trial; RCOT, Randomized Crossover Trial; Acute, < 1 month; Subacute, 1–3 months; Chronic, >3 months*.

Most commonly, authors initiated TX immediately following tDCS. Conversely, four studies employed tDCS throughout the entire duration of the intervention with positive results. Fifteen studies used behavioral measures only, while 8 studies used behavioral and physiological measures, with one study using physiological measures alone. The type of outcome measure is important as tDCS can differentially affect voluntary control outcomes, such as improvement in dexterity, without improvement in strength (Au-Yeung et al., 2014). Further, motor evoked potentials as measured by TMS are often included as an adjunctive measurement tool alongside voluntary control measures (Cortes et al., 2012) as they provide a physiologic measure of corticospinal tract integrity, a crucial pathway in motor control.

The vast majority of studies reported positive UE motor outcomes, which contrasts results of the 2013 Cochrane review (Elsner et al., 2013). The overall consensus of studies favored anodal stimulation over other montages, both before (Cha et al., 2014; Kim et al., 2014; Bolognini et al., 2015; Kasashima-Shindo et al., 2015) and during rehabilitative treatment (Cho and Cha, 2015; Dumont et al., 2015; Rocha et al., 2015). Sixteen studies using anodal stimulation reported a positive effect, with only two studies (Au-Yeung et al., 2014; Triccas et al., 2015) reporting a null effect. Interestingly, the two studies reporting a null effect of anodal stimulation (Au-Yeung et al., 2014; Triccas et al., 2015) delivered an intensity of 1 mA, while the majority of other studies with positive effects administered 1.5–2 mA, indicating a potential beneficial effect of a higher stimulation intensity. Eight studies using bilateral stimulation noted a positive treatment effect, with only one study (Middleton et al., 2014) reporting no effect. In total, six studies employed cathodal stimulation and had positive results, with only Fusco et al. and Rocha et al. reporting no effect (Fusco et al., 2014; Rocha et al., 2015). Overall, most studies that administered tDCS *concurrent* to motor therapy (as opposed to before, after, or not including a therapy component) reported positive results, supporting the hypothesis that tDCS may be most effective and motor recovery most pronounced when it is combined with physical practice. One exception to this finding is the results of a trial administering tDCS concurrent to robotic training (Triccas et al., 2015); however, the heterogeneity of the sample (both in chronicity, impairment level and nature of concurrent therapies), as well as the small sample size, may have contributed to the diminished efficacy reported in this single trial. While the results of these trials are largely promising, the possibility of publication bias should be acknowledged, as studies with significant findings are more likely to be accepted and published, which would cause the literature to be skewed in direction of positive results.

Despite these highly positive findings, there was considerable variance in the nature of behavioral therapy (TX) (e.g., tasks performed, repetitions, duration), and subject characteristics (e.g., affected hemisphere, cortical, or subcortical). It is likely that a number of therapeutic approaches will be effective when combined with tDCS. Nonetheless, standardizing the UE training and minimizing individual differences in the sample is necessary in future work to permit scientists to discern the most important TX ingredients. For example UE robotics enable the TX to be well-defined and consistent across subjects. Additionally, the combination of tDCS with this efficacious form of UE motor training (Lo et al., 2010) may result in enhancement of corticospinal excitability (Edwards et al., 2009) and increased voluntary motor control (Giacobbe et al., 2013).

In addition to motor tasks, the integration of cognitively-loaded behavioral tasks alongside motor training has positively affected UE outcomes. For instance, a brain-computer interface with which subjects plan and execute movements while incorporating attention, pre-frontal decision-making and problem solving significantly improves UE outcomes (Kasashima-Shindo et al., 2015), as has the use of virtual reality (Lee and Chun, 2014; Viana et al., 2014), mirror therapy (Cho and Cha, 2015), and imitation of hand gestures (Bolognini et al., 2015). Congruent with incorporating pre-frontal attention skills during motor training, Watanabe et al. recently reported that providing an executive function task during a stepping exercise reduced incidence of falls (Watanabe et al., 2015). Given the role of the pre-frontal cortex in goal-directed behaviors and initiation of movement, motor interventions that facilitate executive functioning skills may capitalize on the recruitment of top-down processing. Thus, future tDCS interventions may incorporate cognitively challenging tasks as a means to augment motor outcomes.

In addition to modulating TX, methods for harmonizing the targeting of cortical areas are currently under investigation. For example, pathophysiology studies suggest that current logic for altering excitability in stroke survivors may be over-simplified, with a need to prescribe stimulation according to individualized neurophysiological or neuroanatomical factors (Stinear et al., 2015; Thickbroom et al., 2015). Moreover, while the bihemispheric technique had produced some successful outcomes (Lindenberg et al., 2010), more recent work has been equivocal (O'Shea et al., 2014; Ang et al., 2015, Table 1). Other techniques to increase precision of targeting include high definition 4 × 1 tDCS (Edwards et al., 2013), which consists of a center active electrode surrounded by 4 return electrodes, and has been shown to lead to longer lasting physiological effects (Kuo et al., 2013). Individualized montages designed using subject-specific neuroanatomical factors from high resolution MRI (Dmochowski et al., 2011, 2013), may provide the most robust targeting in the future.

## Conclusions

tDCS constitutes a promising adjunctive tool to increase post-strike UE motor function. More research is needed to refine variables such as targeting, dosing, individualized pathophysiological bases, optimal training, and optimal timing of training, which may account for subject response variability. Elucidation of these variables could allow for safe, efficacious and potentially widespread integration of tDCS into clinical rehabilitative therapies.

## Author contributions

All authors listed, have made substantial, direct and intellectual contribution to the work, and approved it for publication.

### Conflict of interest statement

The authors declare that the research was conducted in the absence of any commercial or financial relationships that could be construed as a potential conflict of interest.
